# Hydrophobically Modified Gelatin Particles for Production of Liquid Marbles

**DOI:** 10.3390/polym14224849

**Published:** 2022-11-10

**Authors:** Takayuki Takei, Rio Tomimatsu, Takanori Matsumoto, Kamalalayam Rajan Sreejith, Nam-Trung Nguyen, Masahiro Yoshida

**Affiliations:** 1 Department of Chemical Engineering, Graduate School of Science and Engineering, Kagoshima University, 1-21-40 Korimoto, Kagoshima 890-0065, Japan; 2 Queensland Micro- and Nanotechnology Centre, Nathan Campus, Griffith University, 170 Kessels Road, Nathan, QLD 4111, Australia

**Keywords:** liquid marble, gelatin, spreading coefficient, surface tension

## Abstract

The unique properties and morphology of liquid marbles (LMs) make them potentially useful for various applications. Non-edible hydrophobic organic polymer particles are widely used to prepare LMs. It is necessary to increase the variety of LM particles to extend their use into food and pharmaceuticals. Herein, we focus on hydrophobically modified gelatin (HMG) as a base material for the particles. The surface tension of HMG decreased as the length of alkyl chains incorporated into the gelatin and the degree of substitution (DS) of the alkyl chains increased. HMG with a surface tension of less than 37.5 mN/m (determined using equations based on the Young–Dupré equation and Kaelble–Uy theory) successfully formed LMs of water. The minimum surface tension of a liquid in which it was possible to form LMs using HMG particles was approximately 53 mN/m. We also showed that the liquid-over-solid spreading coefficient $S_{L/S}$ is a potential new factor for predicting if particles can form LMs. The HMG particles and the new system for predicting LM formation could expand the use of LMs in food and pharmaceuticals.

## 1. Introduction

Liquid marbles (LMs) are defined as non-sticking millimeter-sized droplets covered with micro- or nanoparticles with low surface tension [1,2]. Such constructs are commonly observed in nature. For instance, aphids secrete LMs of honeydew to avoid drowning in the liquid in their nidus [3]. LMs can maintain their sphericity on any solid surface without liquid leakage. Furthermore, researchers have confirmed that LMs can be manipulated by external forces, such as gravity, electrical, and magnetic forces [4,5]. Methods to separate the particles from LMs are also reported [6]. The unique properties and morphology of LMs make them attractive for various applications, including microreactors, sensors, energy, foods, and pharmaceuticals [2,7,8,9,10]. In our previous study, we used LMs to fabricate well-designed core–shell capsules [11].

Non-edible hydrophobic organic polymer particles are widely used for the preparation of LMs. However, for food and pharmaceutical applications, it is necessary to use LM particles derived from edible materials. To date, acetylated cellulose and soybean wax have been used as the base materials for LMs particles [12,13]. It is necessary to increase the variety of particles to further extend the use of LMs into food and pharmaceuticals.

The present study aimed to evaluate the potential of hydrophobically modified gelatin (HMG) as a base material for LM particles. Gelatin is an edible natural polymer obtained through the hydrolysis of collagen. Owing to its high safety and extremely low cytotoxicity, the protein has been used extensively in both food and pharmaceuticals. To further extend the use of the protein to various applications, we previously synthesized HMG by incorporating hydrophobic alkyl chains into gelatin molecules to increase their hydrophobicity [14]. Our research showed that the HMG formed more physically stable crosslinked hydrogels than unmodified gelatin, and that the hydrogels were useful as a carrier of both charged hydrophilic drugs and non-charged hydrophobic drugs. Herein, we hypothesized that the modification of gelatin with hydrophobic alkyl groups would decrease the surface tension of the protein, increasing its potential as a base material for LM particles.

In the present study, we first prepared HMGs with alkyl chains of various lengths and determined their surface tension. We then assessed the usefulness of a new quantitative method for predicting whether particles such as HMG will form LMs. Finally, we examined the stability of LMs prepared from HMC particles.

## 2. Materials and Methods

### 2.1. Synthesis of HMG

HMG was synthesized according to the method described in our previous report [14]. Gelatin (type B) (Sigma-Aldrich Co., St. Louis, MO, USA) was dissolved in phosphate-buffered saline (pH 7.4, 200 mL, 3% (*w*/*v*)) by heating. Ethanol (99.8% (*v*/*v*)) was added to the solution in a volume ratio of 5 (ethanol) to 7 (polymer solution), and the resulting solution was cooled to room temperature. After mixing with fatty aldehydes (butanal (C4), hexanal (C6), octanal (C8), or dodecanal (C12); Wako Pure Chemical Industries, Ltd., Osaka, Japan) dissolved in a small amount of ethanol, the solution was gently stirred for 6 h to form imine bonds between the amino groups of gelatin and the aldehyde groups of the fatty aldehydes. Table 1 shows the feed molar ratios of fatty aldehydes to free amino groups in the gelatin. 2-methylpyridine borane (1.7 g) (Junsei Chemical Co. Ltd., Tokyo, Japan), a reductant, was then added to the solution, which was stirred for a further 24 h for reductive amination. HMG was precipitated by adding a large amount of ethanol, collected by centrifugation, and lyophilized. The degree of substitution (DS) values of the alkyl groups were estimated by determining the number of unmodified amino groups in the HMG using the trinitrobenzene sulfonic acid method [15]. The solid HMG was ground to obtain fine particles.

### 2.2. Determination of Interfacial Tension

The surface tension of each liquid was measured using the Wilhelmy plate method with a surface tensiometer (CBVP-A3, Kyowa Interface Science Co., Ltd., Saitama, Japan). During the measurement, the temperature was maintained at 20 ± 1 °C.

A thin film of HMG on a glass slide was first prepared to determine the surface tension of solid HMG ($\gamma_{S}$). Briefly, HMG was dissolved in dimethyl sulfoxide (DMSO) at a concentration of 10% (*w*/*v*). The solution was applied to a glass slide by spin-coating, then dried to form a thin film of HMG. The surface tension of the solid HMG was calculated from the contact angle of the HMG film with two probe liquids (double-distilled water and diiodomethane) using the following two equations based on the Young–Dupré equation and Kaelble–Uy theory [16,17,18]:

(1) $$\gamma_{L}\left(1+\cos\theta \right)=2\left(\sqrt{\gamma_{L}^{d}\;\gamma_{S}^{d}}+\sqrt{\gamma_{L}^{p}\;\gamma_{S}^{p}}\right)$$

(2) $$\gamma_{S}=\gamma_{S}^{d}+\gamma_{S}^{p}$$

where *θ* is the static contact angle of a probe liquid drop on the solid surface, $\gamma_{L}$ is the surface tension of the probe liquids (double-distilled water: 72.8 mN/m, diiodomethane: 50.8 mN/m), $\;\gamma_{S}^{d}$ is the dispersive component of the solid, $\;\gamma_{S}^{p}$ is the polar component of the solid, $\gamma_{L}^{d}$ is the dispersive component of the liquid (double-distilled water: 21.8 mN/m, diiodomethane: 48.5 mN/m), and $\gamma_{L}^{p}$ is the polar component of the liquid (double-distilled water: 51.0 mN/m, diiodomethane: 2.3 mN/m). The static contact angles (droplet volume: 1 µL) were measured using a contact angle meter (DMe-200; Kyowa Interface Science Co., Ltd.). At least 10 measurements were made for each liquid.

The interfacial tension between solid and liquid ($\gamma_{\mathrm{LS}}$) was calculated from the contact angle measured above using Young’s equation:

(3) $$\gamma_{\mathrm{LS}}=\gamma_{S}-\gamma_{L}\cos\theta$$

where $\gamma_{S}$ was calculated from Equations (1) and (2).

### 2.3. Preparation of LMs

The LMs were prepared by adding 50 µL of liquid dropwise to the HMG powder, and gently rolling it. To evaluate their stability, the LMs were gently placed on the surface of double-distilled water in a glass petri dish using a spatula, and the time required for them to collapse was determined [19].

### 2.4. Sphericity of the LMs

The sphericity of a capsule was defined by the following equation:

Sphericity of capsule = (shortest diameter of LMs/longest diameter of LMs) × 100.

## 3. Results and Discussion

**Figure 1:**
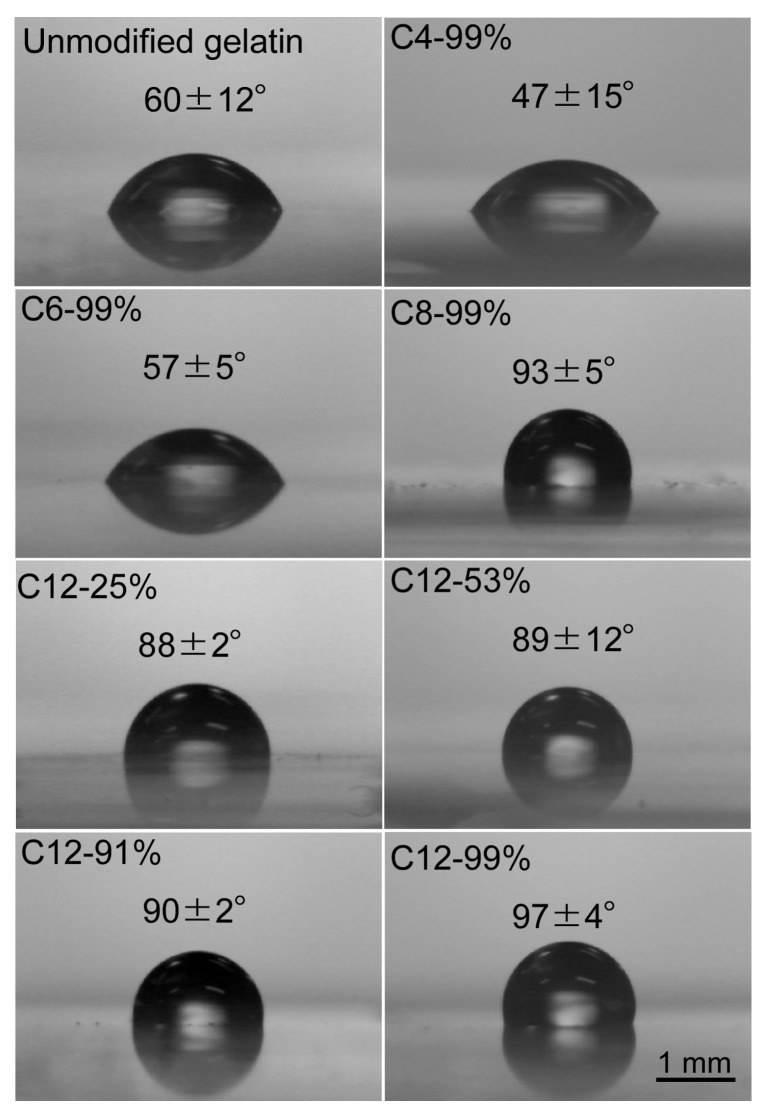
Contact angles of double-distilled water on a hydrophobically modified gelatin (HMG) film.

**Figure 2:**
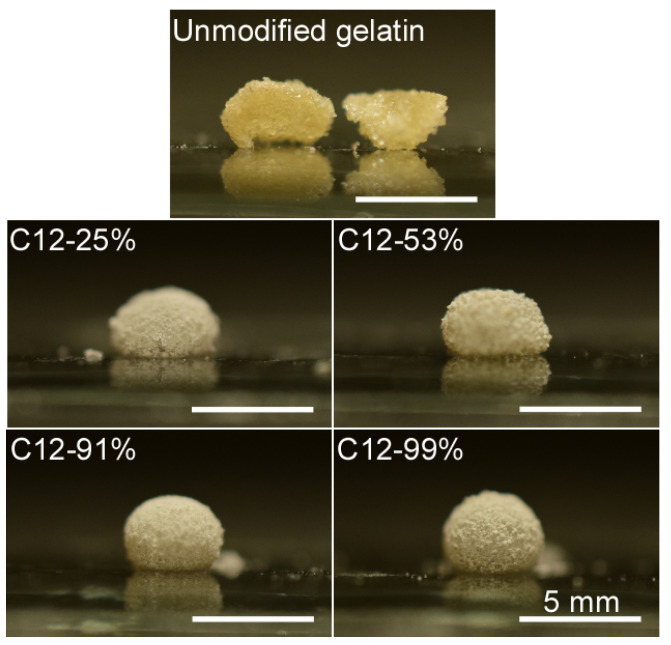
Formation of liquid marbles (LMs) of water from hydrophobically modified gelatin (HMG) particles.

**Figure 3:**
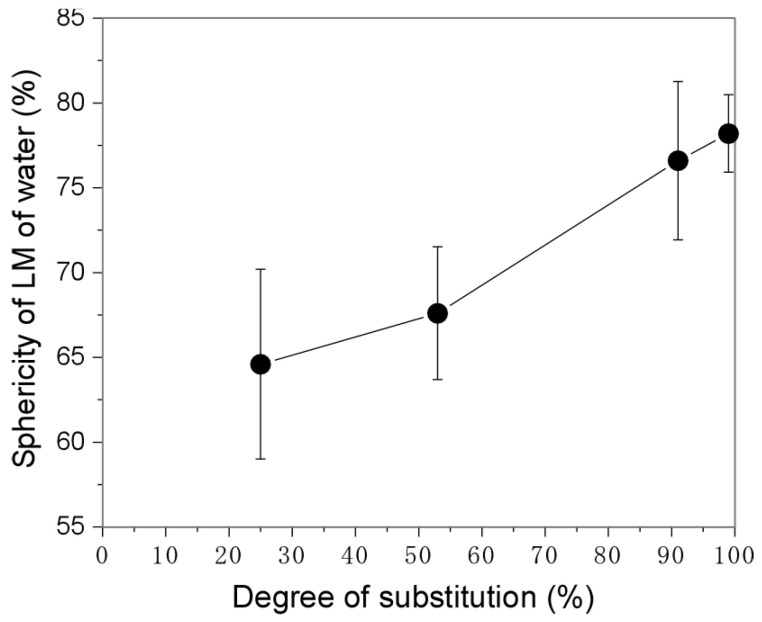
Sphericity of liquid marbles (LMs) of water using C12-25%–C12-99% particles.

The HMG was synthesized according to the method described in our previous report [14]. First, we determined the effects of the length (C4, C6, C8, and C12) and DS of the alkyl groups on the hydrophobicity of the HMG (Table 1). For the C4–C8 alkyl chains, we used an HMG with a DS of 99%. For the C12 alkyl chain, the DS was varied from 25% to 99%. As expected, the static contact angle of double-distilled water on the HMG thin film increased as both the lengths of the alkyl chains and the DS increased (Figure 1). Significant increases in the water contact angle were observed for C8 and C12 alkyl chains. Next, the surface tension of solid HMG was determined on the basis of the Young–Dupré equation and Kaelble–Uy theory. The dispersive component ($\gamma_{S}^{d}$) remained almost unchanged (Table 1). However, the polar component ($\gamma_{S}^{p}$) decreased as both the lengths of the alkyl chains and the DS increased, which was partially due to the consumption of polar amino groups to form amide bonds. The decrease in the polar component resulted in a decrease in the surface tension of the HMG. It has been reported that the stability of LMs depends on the size of the particles used [20]. Therefore, the size of the particles was roughly set by grinding the HMG before preparing the LMs of water (Table 1). The unmodified gelatin, C4-99%, and C6-99% particles were well-wetted with water and formed agglomerates (not LMs) (Figure 2 and Table 2). It should be noted that the other HMGs successfully formed LMs. The sphericity of the LMs increased as the DS of the alkyl chain increased (Figure 3). The sphericity of LM depends on the difference between surface tensions of the liquid and the particles [21]. Decrease in the surface tension of HMG with increased DS would increase interfacial tension between the liquid and the particles, resulting in an increase in sphericity of LM. These results show that C8-99% and C12-25%–C12-99% derived from edible gelatin are promising for the formation of LMs of water that is widely used in food and pharmaceutical fields.

As described in the Introduction, acetylated cellulose and soybean wax have been used as the base materials for edible LMs particles [12,13]. Soybean wax typically has high hydrophobicity. On the other hand, the cellulose derivative was chemically modified (acetylation) to increase its hydrophobicity as HMG was. C12-99% HMG has almost the same low surface tension as the acetylated cellulose that was maximumly modified (Table 2). Another feature of HMG is that the base material is protein, indicating HMG is useful in applications where proteins are preferred as LM particles.

It has been reported that the solid-over-liquid spreading coefficient ($S_{S/L}$), which is defined in the following equation, provides a quantitative method for predicting whether given particles can form LMs [22,23,24,25]:

(4) $$S_{S/L}=-2\gamma_{S}+2{\left(\gamma_{L}^{d}\;\gamma_{S}^{d}\right)}^{\frac{1}{2}}+2{\left(\gamma_{L}^{p}\;\gamma_{S}^{p}\right)}^{\frac{1}{2}}$$

A positive $S_{S/L}$ means that the particles tend to spread well over the water, and hence form LMs. Some researchers have reported that LMs are formed when the $S_{S/L}$ is above a certain positive value. For example, Zhou et al. acetylated cellulose particles to increase their hydrophobicity, and reported that only particles with a DS of 0.39 or more formed LMs of water [12]. The $S_{S/L}$ values of the acetylated cellulose particles are presented in Table 2. We determined whether the theory could be applied to our HMG particles. Table 2 presents the $S_{S/L}$ values for HMG when the liquid is water. Contrary to expectations, the $S_{S/L}$ values were positive when the conditions were such that LMs could not be formed. These results show that the conventional theory cannot be applied to HMG particles to predict the formation of LMs. To establish a new theory that is applicable to HMG particles, we focused on the liquid-over-solid (not the solid-over-liquid) spreading coefficient $S_{L/S}$ defined by the following equation:

(5) $$S_{L/S}=-2\gamma_{L}+2{\left(\gamma_{L}^{d}\;\gamma_{S}^{d}\right)}^{\frac{1}{2}}+2{\left(\gamma_{L}^{p}\;\gamma_{S}^{p}\right)}^{\frac{1}{2}}$$

Aussillous et al. reported that, from the thermodynamic perspective, LM-forming particles lower their own interfacial/surface-free energy (ΔG<0) by adsorption on the liquid droplet surface [26]. Therefore, the adsorption of the particles on the surface of a liquid droplet is a spontaneous process. However, if the particles are “excessively” wetted by the liquid, they do not form LMs (as shown in unmodified gelatin in Figure 2). In other words, to prepare LMs, the particles must be resistant to “excessive” wetting by the liquid. Therefore, we defined the spreading coefficient $S_{L/S}$. Theoretically, a negative $S_{L/S}$ means that the particles tend not to be wetted by the liquid, and hence form LMs. Table 2 shows the $S_{L/S}$ for each HMG particle when the liquid is water. The particles that were able to form LMs of water had $S_{L/S}$ values of less than −32.5. Our calculations show that particles of acetylated cellulose would have to have an $S_{L/S}$ of less than −22.5 to form LMs of water (Table 2). These data indicate that the threshold value of $S_{L/S}$, which determines whether LMs can be formed, seems to be −32.5 to −22.5, and $S_{L/S}$ is a potential new factor for predicting LM formation.

It is difficult to prepare LMs from a liquid with a smaller surface tension than that of water because the difference between the surface tension of the liquid and the particles becomes smaller [12]. Herein, we examined the minimum surface tension of the liquid for LM formation using C12-99% HMG particles. As shown in Table 3, the minimum surface tension was approximately 53 mN/m. Finally, we measured the time it took for the LMs to rupture when placed on the surface of a pool of water. LMs of water coated with C12-99% particles were stable on water even more than 24 h, showing its usefulness in practical application. As the surface tension of the liquid decreased, the time to rupture also decreased.

## 4. Conclusions

In the present study, we investigated HMG as a potential base material for the preparation of LM particles. An HMG with a surface tension of less than 37.5 mN/m was capable of forming LMs of water. The minimum surface tension of a liquid whereby LMs were formable using HMG particles was approximately 53 mN/m. We showed that the liquid-over-solid spreading coefficient, $S_{L/S}$, is a potential new factor for predicting whether the given particles can form LMs. The HMG particles and the new system for predicting LM formation could expand the use of LMs in food and pharmaceuticals.

## Figures and Tables

**Table 1:** Characteristics of hydrophobically modified gelatin (HMG).

Name	Alkyl Chain Length of Fatty Aldehyde	Feeding Molar Ratios of Fatty Aldehyde to Amino Groups of Gelatin (%)	Degree of Substitution (%)	$\gamma^{d}$	$\gamma^{p}$	γ $(=\gamma^{d}$ $+\;\gamma^{p})$	Particle Size (μm)
Unmodified gelatin	—	—	—	32.1 ± 3.5	16.8 ± 8.2	48.8 ± 5.3	30 ± 12
C4-99%	C4	500	99	29.6 ± 3.4	25.1 ± 9.9	54.6 ± 6.8	48 ± 32
C6-99%	C6	500	99	30.8 ± 2.5	18.6 ± 3.2	49.4 ± 4.1	61 ± 38
C8-99%	C8	500	99	36.4 ± 1.6	1.1 ± 0.8	37.5 ± 0.9	33 ± 16
C12-25%	C12	50	25	35.7 ± 0.1	1.9 ± 0.4	37.6 ± 0.3	56 ± 45
C12-53%	C12	100	53	35.3 ± 0.4	1.7 ± 0.1	37.0 ± 0.4	48 ± 33
C12-91%	C12	200	91	35.4 ± 0.1	1.4 ± 0.4	36.9 ± 0.3	51 ± 28
C12-99%	C12	500	99	34.5 ± 4.2	0.6 ± 0.4	35.1 ± 4.1	44 ± 31

**Table 2:** Relationship between the ability of hydrophobically modified gelatin (HMG) to form liquid marbles (LMs) of water and spreading coefficients.

		Formation of Water LM ^a^	$S_{S/L}$	$S_{L/S}$
Unmodified gelatin		–	14.1	−36.3
HMG	C4-99%	–	14.0	−25.7
	C6-99%	–	14.8	−32.5
	C8-99%	+	−4.8	−76.0
	C12-25%	+	0.3	−70.0
	C12-53%	+	0.0	−71.5
	C12-91%	+	−1.1	−73.0
	C12-99%	+	−7.1	−82.3
Acetylated cellulose	0 (60.7 mN/m) ^b,c^	– ^b^	3.1 ^b^	−21.1
	0.14 (58.5 ± 3.7 mN/m) ^b,c^	– ^b^	6.2 ^b^	−22.5
	0.39 (54.6 ± 3.3 mN/m) ^b,c^	+ ^b^	8.9 ^b^	−27.5
	1.26 (49.9 ± 2.5 mN/m) ^b,c^	+ ^b^	9.5 ^b^	−42.1
	2.61 (38.6 ± 2.1 mN/m) ^b,c^	+ ^b^	10.1 ^b^	−58.5

^a^ +: LM formed, –: LM did not form. ^b^ From [12]. ^c^ Values outside and inside parentheses represent the degree of substitution (DS) of the acetyl group (maximum: 3.0) and the surface tension, respectively.

**Table 3:** Formation of liquid marbles (LMs) from various liquids with different surface tensions using C12-99% hydrophobically modified gelatin (HMG).

Liquid		Formation of LM ^a^	Time until LM on Water Collapsed
Component	Surface Tension (mN/m)		
Water	72.8	+	>24 h
Glycerin	63.4	+	7.8 ± 2.3 min
Formamide	58.2	+	2.8 ± 0.8 min
Glycerin (25%)/DMSO (75%) ^b^	57.0 ± 0.2 ^c^	+	42 ± 24 sec
Glycerin (50%)/DMSO (50%) ^b^	52.6 ± 0.3 ^c^	+	<0.1 sec
Diiodomethane	50.8	–	–
DMSO	45.1	–	–

^a^ +: LM formed, –: LM did not form. ^b^ volume basis. ^c^ The data were obtained experimentally.

## Data Availability

Data presented in this study are available on request from the corresponding author.
